# Experimental murine myopia induces collagen type Iα1 (*COL1A1*) DNA methylation and altered *COL1A1* messenger RNA expression in sclera

**Published:** 2012-05-30

**Authors:** Xiangtian Zhou, Fengtao Ji, Jianhong An, Fuxin Zhao, Fanjun Shi, Furong Huang, Yuan Li, Shiming Jiao, Dongsheng Yan, Xiaoyan Chen, JiangFan Chen, Jia Qu

**Affiliations:** 1 School of Optometry & Ophthalmology and Eye Hospital, Wenzhou Medical College, Wenzhou, Zhejiang, China; 2 State Key Laboratory Cultivation Base and Key Laboratory of Vision Science, Ministry of Health P.R. China and Zhejiang Provincial Key Laboratory of Ophthalmology and Optometry, Wenzhou, Zhejiang, China; 3 Department of Neurology, Boston University School of Medicine, Boston, MA

## Abstract

**Purpose:**

To investigate whether myopia development is associated with changes of scleral DNA methylation in cytosine-phosphate-guanine (CpG) sites in the collagen 1A1 (*COL1A1*) promoter and messenger RNA (mRNA) levels following murine form deprivation myopia.

**Methods:**

Fifty-seven C57BL/6 mice (postnatal day 23) were randomly assigned to four groups: (1) monocular form deprivation (MD) in which a diffuser lens was placed over one eye for 28 days; (2) normal controls without MD; (3) MD recovery in which the diffuser lens was removed for seven days; and (4) MD recovery normal controls. The DNA methylation pattern in *COL1A1* promoter and exon 1 was determined by bisulfite DNA sequencing, and the *COL1A1* mRNA level in sclera was determined by quantitative PCR.

**Results:**

MD was found to induce myopia in the treated eyes. Six CpG sites in the promoter and exon 1 region of *COL1A1* were methylated with significantly higher frequency in the treated eyes than normal control eyes (p<0.05), with CpG island methylation in MD-contralateral eyes being intermediate. Consistent with the CpG methylation, scleral *COL1A1* mRNA was reduced by 57% in the MD-treated eyes compared to normal controls (p<0.05). After seven days of MD recovery, CpG methylation was significantly reduced (p=0.01). The methylation patterns returned to near normal level in five CpG sites, but the sixth was hypomethylated compared to normal controls.

**Conclusions:**

In parallel with the development of myopia and the reduced *COL1A1* mRNA, the frequency of methylation in CpG sites of the *COL1A1* promoter/exon 1 increased during MD and returned to near normal during recovery. Thus, hypermethylation of CpG sites in the promoter/exon 1 of *COL1A1* may underlie reduced collagen synthesis at the transcriptional level in myopic scleras.

## Introduction

Myopia is the most common eye disorder in the world, and its prevalence is estimated to be 33% in some Western countries [1,2]. It is especially high, 65 to 88%, in students from Asian regions and countries, including Hong Kong [3-5], Taiwan [6], and Singapore [7]. However, the mechanism by which myopia develops has not been fully clarified.

Several lines of experimental evidence strongly suggest that the pathological changes in the sclera of myopic eyes can be associated with reduced synthesis and increased degradation of type I collagen [8]. Each monomeric unit of type I collagen protein is a heterotrimer composed of two type I alpha 1 (COL1A1) and one type I alpha 2 (COL1A2) chains. The gene for the major component of type I collagen (*COL1A1*) [9], is located on human chromosome 17 (17q21.33), within the high myopia candidate locus MYP5 (17q21–22) [10-12]. Several studies have focused on its expression during myopia [13-15]. In the tree shrew, expression of collagen type I messenger RNA (mRNA) is reduced in the sclera of myopic eyes and increases to normal levels during myopia recovery [13-15]. However the mechanism of *COL1A*1 modulation in myopia still remains unclear.

One mechanism of gene expression regulation is mediated by DNA methylation of cytosine-phosphate-guanine (CpG) sites within promoters. This process can generally lead to gene silencing, a feature found in several human cancers in which expression of tumor suppressor genes is inhibited [16,17]. In contrast, the hypomethylation of CpG sites is associated with the overexpression of oncogenes within cancer cells [18]. DNA methylation is controlled by an array of DNA methylation transferases and demethylation enzymes. The promoter region of *COL1A1* contains CpG islands [19], and methylation in this region, as well as in exon 1, depresses *COL1A1* gene expression in cultured 3T3 mouse embryo tissue fibroblasts and F9 embryonal carcinoma cells [19]. Suppression of *COL1A1* gene expression is associated with increased DNA methylation after the transformation of normal human lung fibroblasts by Simian vacuolating virus 40 (SV40) [20]. However, there have been no reports on changes in *COL1A1* methylation or that of other genes in the development of myopia. In this study, we used the experimental mouse model of myopia to evaluate the methylation status of CpG sites in the promoter and exon 1 region of *COL1A1* in the scleras of myopic and control eyes. We also correlated the DNA methylation pattern with the expression of *COL1A1* mRNA during the onset of myopia.

## Methods

### Development of form-deprivation myopia in mice

All animals were obtained from the animal breeding unit at Wenzhou Medical College and raised in standard mouse cages with a 12 h:12 h light-dark cycle. The study was approved by the Animal Care and Ethics Committee at Wenzhou Medical College (Wenzhou, China). The experiments were conducted in accordance with the ARVO Statement for the Use of Animals in Ophthalmic and Vision Research.

Four groups of 23-day-old C57BL/6 mice were included in the study: (1) A monocular deprivation (MD) group (n=28) was form deprived for four weeks, from 23 to 51 days of age. This was achieved by the placement of a light-diffusing lens over a randomly chosen eye as Schaeffel et al. [21] described. (2) An age-matched normal control group (n=14) was maintained free of form deprivation for the same four-week period. (3) A separate MD group (n=10) was allowed to recover by removal of the diffuser lens for seven days (days 51–58) after the four weeks of form deprivation. (4) Finally, another age-matched normal group (n=5) was established for the MD mice that were allowed to recover for seven days. These mice were similar to the first normal control group in that neither eye was form deprived.

Measurements for refraction and ocular dimensions at the beginning and end of the treatment periods were taken as described below.

#### Refraction

The refractive state was measured in a dark room with an eccentric infrared photorefractor as previously described, which was calibrated according to a published procedure [22,23]. Briefly, the mouse was gently restrained by holding its tail and positioning it on a small stage in front of the photoretinoscope. On-axis measurements were recorded when the Purkinje image was present in the center of the pupil [21]. The data were then recorded using software designed by Schaeffel et al. [21]. Measurements were repeated at least three times for each eye.

#### Ocular dimensions

Ocular dimensions, including anterior chamber depth, lens thickness, vitreous chamber depth, and axial length were measured by real-time optical coherence tomography using a custom-built optical coherence tomography instrument [24]. Anterior chamber depth was defined as the distance from the posterior surface of the cornea to the anterior surface of the lens. Axial length was defined as the distance between the anterior surface of the cornea and the vitreous-retinal interface. Each eye was scanned three times.

#### Corneal curvature measurement

Corneal curvature was measured with a keratometer (Topcon OM-4; Topcon Corp., Tokyo, Japan) that was modified by mounting a +20.0-diopter (D) aspherical lens as previously described [22,23]. Each eye was measured three times to obtain a mean value.

### DNA isolation

Mice were sacrificed by an overdose of pentobarbitone sodium. Immediately after removal of the diffuser, the eyes were enucleated and dissected to obtain the sclera free of other tissues. The separated sclera was immediately stored in liquid nitrogen at −80 °C before total DNA was isolated. Due to the small amount of DNA in the scleral tissue, scleras from pairs of eyes were pooled to obtain sufficient DNA for analysis. For the form-deprived eyes, two scleras from the MD-treated (MD-T) eyes were pooled. Scleras from the untreated contralateral eyes (MD-C) of the MD-T mice were also pooled. Scleras from treated eyes that were allowed to recover for seven days were pooled as the MD-treated-recovery group (MD-R). The contralateral control eyes of that group were pooled as the MD-treated-recovery control (MD-RC) group. The final two groups consisted of scleras from normal control mice at 51 and 58 days of age (NC_51_ and NC_58_, respectively). For these control animals, the two eyes were treated in the same way, i.e., they had no treatment; therefore, the scleras (left and right) were pooled from the same animal rather than from separate animals.

Total DNA was extracted with proteinase K treatment and a phenol-extraction procedure according to standard methods [25]. DNA concentration and purity were determined by spectrophotometry at 260 nm and 280 nm. The A_260_/A_280_ absorbance ratio was consistent at approximately 1.8. An average of 1.2 μg of total DNA was obtained from every scleral pool.

### Bisulfite modification of DNA

Bisulfite modification of DNA was performed using the CpGenome DNA Modification Kit (Millipore, Billerica, MA) following the manufacturer’s directions: 1 μg DNA was denatured in 0.3 M NaOH for 10 min at 37 °C in a final volume of 107 μl. It was then mixed with 550 μl of 3.6 M sodium bisulfite and incubated for 16 h at 50 °C.

After alkaline desulfonation and final desalting, single-stranded uracil-containing reaction products were eluted in 30 μl of buffer composed of 10 mM Tris-HCl and 1 mM EDTA at pH 8.0. Sodium bisulfite was used to convert unmethylated cytosine into uracil. Following PCR amplification, all unmethylated cytosines within a sequence were replaced with thymine (Table 1). Methylated cytosines remained as cytosine following PCR amplification.

**Table 1:** Bisulfite sequence PCR measurement mechanism.

**Sequence**	**Unmethylated DNA**	**Methylated DNA**
Initial sequence	AACTGACGTACTACG	AACTGACmGTACTACmG
Converted sequence	AAUTGAUGTAUTAUG	AAUTGACGTAUTACG
PCR product sequence	AATTGATGTATTATG	AATTGACGTATTACG

### Primer design and PCR amplification of bisulfite-treated DNA

Bisulfite sequencing PCR was based on the indiscriminant amplification of a section of methylated or unmethylated DNA containing CpG sites within the amplicon but not the primer sequence (Table 1). It requires only one set of primers to amplify both methylated and unmethylated DNA, which can then be distinguished by subsequent sequencing. Using the Methyl Primer Express v1.0 (Applied Biosystems, Foster City, CA), PCR primers were designed according to the published DNA sequences of *COL1A1*: Forward, 5′-GTT TAT GTA GAT TTG GGG GGT A-3′; reverse, 5′-AAC TCC CCA AAA TTT AAA ACT T-3. The primers were specially tested using methBLAST. The amplified 447 base pair (bp) fragment was between −247 and +200 in the *COL1A1* promoter and exon 1 region. It contained 19 CpG dinucleotide sites.

PCR amplification of 100 ng bisulfate-treated DNA template was performed in a reaction mixture containing 0.5 μl 20 pM forward and reverse primers, 4 μl of 25 mM Mg^2+^, 10 μl of 5× buffer, 1 μl of 2.50 mM deoxynucleotide triphosphates, 0.25 μl of Go Taq Hot Start Polymerase (Promega, Madison, WI), and 34.25 μl distilled water for a total volume of 50 µl. Amplification conditions included an initial denaturation at 95 °C for 10 min, followed by 40 cycles at 95 °C for 30 s, 55.7 °C for 30 s, and 72 °C for 1 min. The final extension at 72 °C lasted 10 min. The purified PCR products were cloned into plasmid vectors by means of a TOPO TA Cloning Kit (Invitrogen, Carlsbad, CA), and around 30 positive clones were chosen for sequencing. Successful ligations were detected by blue-white selection, and positive clones were selected for PCR using the same amplification conditions described above. Since there were 19 CpG sites in the 5′ promoter region of *COL1A1*, we analyzed 19 sites × 5 samples per group=95 CpG sites per experimental group. The percentage of methylated CpGs was calculated by the number of methylated CpGs divided by the total number of CpGs analyzed.

Many transcription factors may bind to the DNA sequence of the amplified fragment, the online software of P-Match 1.0 was used to predict transcription factor binding sites.

### RNA isolation

Scleras were isolated and pooled as described above. To avoid mRNA degradation, the scleras were placed immediately into room-temperature RNA Later (Ambion, Foster City, CA). The RNA Later was then removed after remaining at 4 °C overnight, and the scleras were stored at −80 °C for later use.

Total RNA was extracted using the RNeasy Fibrous Tissue Mini Kit (Qiagen, GmbH, Hilden, Germany) at room temperature. Tissue samples were pooled as described above (pooled MD-T eyes, n=9; pooled MD-C eyes, n=9; pooled normal control eyes, n=9). RNA concentration and purity were determined by spectrophotometry at 260 nm and 280 nm. The A_260_/A_280_ absorbance ratio was consistently about 1.9, indicating high purity of RNA. An average of 1 μg of total RNA was obtained from each of the pooled scleras. To remove contaminating genomic DNA, 1 μg of total RNA was treated with 1 U RNase free DNase I (Promega, Madison, WI) at 37 °C for 30 min and then heated with 1 μl stop solution (Promega) at 65 °C for 10 min.

### Quantitative PCR

Single-strand cDNA was synthesized from 400 ng RNA in 20 µl of reaction volume using the preamplification system M-MLV Reverse Transcriptase (Promega). After reverse transcription, the *COL1A1* mRNA level was measured by real-time reverse transcriptase (RT)-PCR analysis (Power SYBR Green PCR Master Mix; Applied Biosystems) [26]. Primers were designed using Primer Express 3.0 software (Applied Biosystems) and amplified 100 bp to 150 bp cDNA fragments (Table 2). The mouse 18S rRNA gene was used as an internal control based on its constant level of expression among the different groups [27].

**Table 2:** Quantitative PCR gene primer pairs.

**Gene name**	**Forward Primers (5′-3′)**	**Reverse Primers (5′-3′)**	**Length (bp)**
*COL1A1*	GAGAGCGAGGCCTTCCCGGA	GGGAGCCAGCGGGACCTTGT	131
*18S rRNA*	CGGACACGGACAGGATTGAC	TGCCAGAGTCTCGTTCGTTATC	124

*COL1A1*: collagen type Iα1; 18S rRNA was the housekeeping gene.

Quantitative PCR was performed with 2.5 nM primers (ABI 7500; Applied Biosystems) and 1 μl of cDNA in a 15 μl reaction for 40 cycles under the following conditions: 50 °C for 2 min and 95 °C for 10 min, followed by 40 cycles of amplification at 95 °C for 15 s and 60 °C for 60 s. All experiments were performed in duplicate.

The expression level of *COL1A1* mRNA was normalized to that of an internal control 18S rRNA [27]. We used the relative expression level to indicate the fold change between different groups of eyes by using the equation of 2^−ΔΔCt^, where:

ΔΔCt = (CTCOL1A1 - CT18S rRNA) MD-T/MD-C/NC51 - (CTCOL1A1 - CT18S rRNA) NC51

### Statistical analysis

Statistical analyses were performed using the Statistical Procedures for the Social Sciences (SPSS 13.0, SPSS, Chicago, IL). Descriptive statistics were calculated as means and standard error. Statistical differences between groups were calculated by independent sample *t* test. Differences of biometric parameter between the MD-T eyes and the MD-C eyes in the same group were calculated by paired sample *t* test and differences of biologic parameter between the MD-T eyes and the MD-C eyes in the same group were calculated by independent sample *t* test. A p value <0.05 was considered to be statistically significant.

## Results

### Confirmation that form deprivation induces myopia

**Figure 1:**
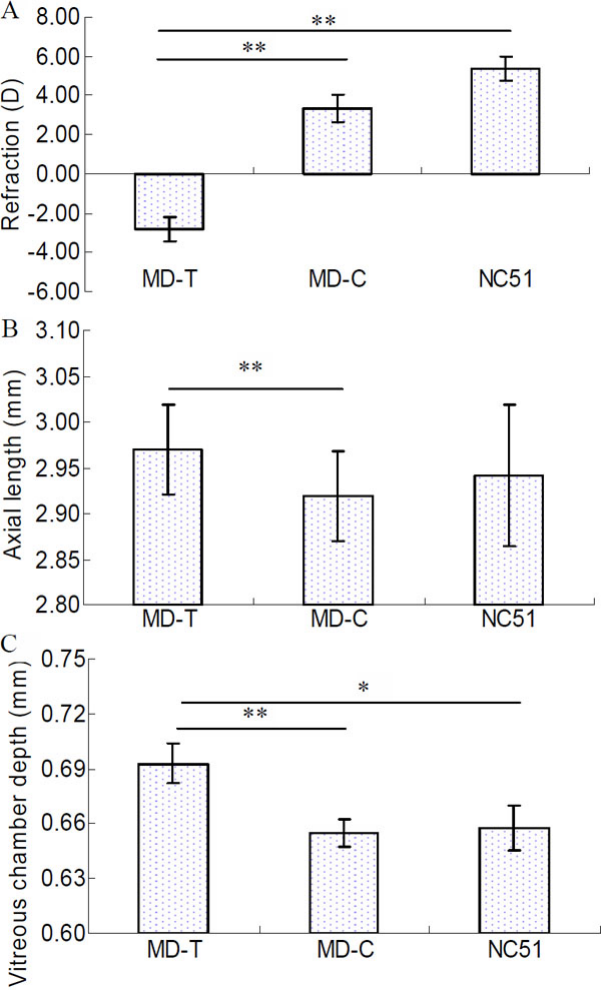
Ocular refraction parameters of mice for quantitative PCR in monocular deprived and control eyes. **A**: Eyes treated by monocular deprivation (MD-T, n=18) for 28 days were significantly more myopic than were contralateral control (MD-C, n=18) and age-matched normal control (NC_51_, n=9) eyes. **B**: The MD-T eyes also exhibited significantly greater axial length than did the MD-C eyes, but not the NC_51_ eyes. **C**: Differences in the vitreous chamber depths among the treated eyes and contralateral control eyes compared to age-matched normal control eyes (NC_51_) were significant, *, p<0.05, **, p<0.01. All error bars in figures show the standard error (SE).

There were no significant differences in refraction or axial length among all groups before the experiment. Additionally, there were no significant differences in refraction or axial length between the two eyes of the same animal (p=0.24 and 0.62, respectively, paired sample *t* test). After 28 days of form deprivation, refractions for the MD-T eyes and MD-C eyes were −2.81±0.63 D and 3.35±0.70 D, respectively (paired *t* test, p<0.001, Figure 1A). Refraction in the MD-T eyes was also significantly different from NC_51_ eyes, 5.39±0.63 D (independent sample *t* test, p<0.001, Figure 1A). The axial lengths for the MD-T eyes and MD-C eyes were 2.97±0.05 mm and 2.92±0.05 mm, respectively (paired *t* test, p<0.001, Figure 1B); however, there were no significant differences in the axial lengths between MD-T eyes and MD-C eyes (2.94±0.08 mm). The vitreous chamber depth for the MD-T eyes, MD-C eyes, and NC_51_ eyes were 0.69±0.01 mm, 0.66±0.01 mm, and 0.65±0.01 mm, respectively. The MD-T vitreous depth was significantly greater than in the MD-C (paired *t* test, p<0.01, Figure 1C) and NC_51_ eyes (independent sample *t* test, p<0.05, Figure 1C). The corneal curvature, anterior chamber depth, and lens thickness were not significantly different when MD-T eyes were compared to MD-C and NC_51_ eyes. Furthermore, there were no significant differences in refraction, axial components, or corneal curvature between MD-C eyes and NC_51_ eyes.

### DNA methylation of the *COL1A1* promoter in the monocular form deprivation (MD) groups

**Figure 2:**
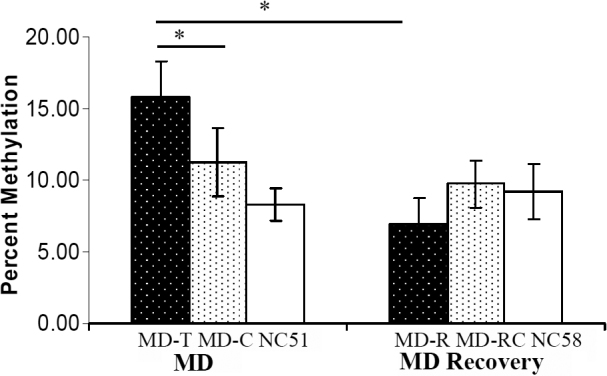
Proportion of sites that were methylated in the proximal promoter and a portion of exon 1. **A**: Form-deprived, contralateral control, and normal control eyes after four weeks of monocular form deprivation. **B**: Form-deprived, contralateral control, and normal control eyes after one week of recovery following four weeks of monocular deprivation (MD). Detailed maps of cytosine-phosphate-guanine (CpG) sites in the proximal promoter and first exon are shown. The beads in the horizontal lines illustrate the CpG sites, and the color of each indicates the corresponding degree of methylation: gray, 0–0.1; blue, 0.1–0.2; green, 0.2–0.3, red, >0.3. MD-T: monocular deprivation-treated eyes, MD-C: contralateral control eyes, NC: age-matched normal control eyes, Numbers: sample IDs.

**Figure 3:**
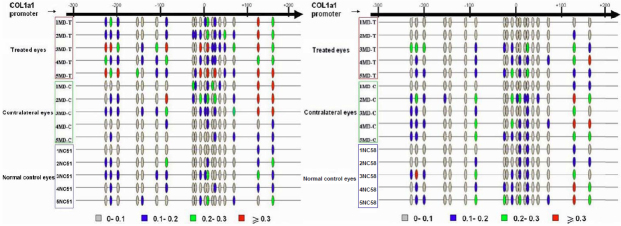
Total DNA methylation in the monocular deprivation, monocular deprivation recovery, and control groups. Methylation of the cytosine-phosphate-guanine (CpG) sites in the monocular deprivation–treated (MD-T) eyes was significantly greater than in normal control and recovery eyes. MD-C: MD contralateral control eyes, NC_51_: age-matched normal control eyes; MD-R: after seven days of recovery following four weeks of monocular deprivation, MD-RC: contralateral control eyes after recovery period, NC_58_: age-matched normal control eyes for MD recovery, *, p<0.05.

**Figure 4:**
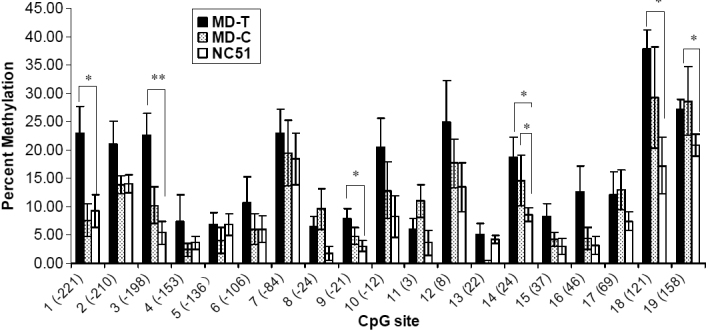
Methylation percentages of cytosine-phosphate-guanine (CpG) sites in the collagen type Iα1 promoter region in scleras of monocular deprivation and control eyes after four weeks of monocular deprivation. Numbers in parentheses on the x-axis are the locations of the cytosine-phosphate-guanine (CpG) sites. Methylation percentages at sites 1, 3, 9, 14, 18, and 19 were significantly greater in monocular deprivation–treated (MD-T) eyes than age-matched normal control (NC_51_) eyes. MD-T: monocular deprivation-treated eyes, MD-C: contralateral control eyes. *, p<0.05, **, p<0.01.

DNA methylation profiles for MD-T, MD-C, and NC_51_ eyes were determined after four weeks of monocular form deprivation (Table 3). In MD-C and NC_51_ eyes, most of the CpG sites exhibited very low levels of DNA methylation, whereas in MD-T eyes, the levels were elevated at most of the sites (Figure 2A). The amount of methylation in MD-T eyes was higher than in MD-C eyes (Figure 3). The methylation percentages of six CpG sites (1, 3, 9, 14, 18, and 19) in MD-T eyes were significantly increased compared to the NC_51_ eyes (Figure 4). In MD-C eyes, the CpG sites were methylated at a level intermediate between the MD-T and NC_51_ eyes (Figure 3). The methylation percentages of four CpG sites (3, 8, 14, and 18) in MD-C eyes tended to increase compared to the NC_51_ eyes, although only site 14 was significantly increased (Figure 4).

**Table 3:** Percentage of DNA methylation in the proximal promoter and a portion of exon 1 of *COL1A1*.

**Num.**	**1**	**2**	**3**	**4**	**5**	**6**	**7**	**8**	**9**	**10**	**11**	**12**	**13**	**14**	**15**	**16**	**17**	**18**	**19**
Position	−221	−210	−198	−153	−136	−106	−84	−24	−21	−12	3	8	22	24	37	46	69	121	158
1MD-T	12.0	21.0	18.0	—	3.0	—	18.0	6.0	3.0	9.0	12.0	21.0	3.0	15.0	3.0	6.0	—	32.0	21.0
2MD-T	11.0	11.0	17.0	—	9.0	—	17.0	11.0	11.0	14.0	—	2.0	6.0	11.0	11.0	26.0	17.0	37.0	29.0
3MD-T	32.0	35.0	21.0	6.0	12.0	24.0	38.0	9.0	6.0	35.0	6.0	41.0	12.0	18.0	15.0	18.0	24.0	47.0	29.0
4MD-T	29.0	16.0	19.0	6.0	1.0	13.0	26.0	—	6.0	13.0	6.0	19.0	1.0	19.0	3.0	—	6.0	29.0	26.0
5MD-T	31.0	22.0	38.0	25.0	9.0	16.0	16.0	6.0	13.0	31.0	6.0	41.0	3.0	31.0	9.0	13.0	13.0	44.0	31.0
1MD-C	11.0	11.0	9.0	3.0	3.0	3.0	26.0	17.0	9.0	9.0	14.0	23.0	—	2.0	6.0	9.0	11.0	37.0	37.0
2MD-C	1.0	16.0	19.0	3.0	1.0	1.0	35.0	19.0	6.0	29.0	19.0	26.0	—	29.0	6.0	1.0	13.0	55.0	45.0
3MD-C	16.0	16.0	16.0	6.0	13.0	16.0	22.0	6.0	3.0	19.0	13.0	19.0	3.0	16.0	6.0	9.0	25.0	34.0	31.0
4MD-C	9.0	9.0	6.0	—	—	3.0	13.0	6.0	6.0	6.0	6.0	19.0	—	9.0	—	—	3.0	3.0	13.0
5MD-C	1.0	17.0	1.0	—	3.0	7.0	1.0	—	—	1.0	3.0	1.0	—	17.0	3.0	3.0	13.0	17.0	17.0
1NC51	6.0	9.0	6.0	—	3.0	3.0	3.0	—	3.0	3.0	3.0	9.0	3.0	9.0	6.0	9.0	6.0	18.0	18.0
2NC51	18.0	14.0	—	6.0	6.0	3.0	23.0	6.0	6.0	3.0	3.0	11.0	6.0	6.0	—	3.0	6.0	14.0	26.0
3NC51	12.0	15.0	12.0	3.0	9.0	15.0	18.0	3.0	3.0	18.0	12.0	24.0	6.0	9.0	6.0	3.0	9.0	3.0	18.0
4NC51	1.0	13.0	3.0	3.0	3.0	3.0	17.0	—	—	1.0	—	1.0	3.0	13.0	3.0	1.0	3.0	17.0	17.0
5NC51	9.0	19.0	6.0	6.0	13.0	6.0	31.0	—	3.0	16.0	—	22.0	3.0	6.0	—	—	13.0	34.0	25.0

Form-deprived, contralateral control eyes after four weeks of monocular form deprivation, and MD age-matched normal control eyes for 51 days. “—” means none detected.

### DNA methylation of the *COL1A1* promoter in the monocular form deprivation (MD) recovery groups

A similar analysis was performed for the MD-R eyes and MD-RC eyes. For each sample, about 30 to 34 DNA clones were analyzed. DNA methylation profiles for MD-R, MD-RC, and NC_58_ eyes were determined after seven days of recovery following four weeks of monocular form deprivation (Table 4 and Figure 2B). In MD-R eyes, the levels of DNA methylation were lower than those seen in MD-T eyes (p<0.01, Figure 3). However, DNA methylation in MD-RC and NC_58_ eyes was not significantly different from that of the MD recovery eyes (Figure 3).

**Table 4:** Percentage of DNA methylation in the proximal promoter and a portion of exon 1 of *COL1A1*.

**Num.**	**1**	**2**	**3**	**4**	**5**	**6**	**7**	**8**	**9**	**10**	**11**	**12**	**13**	**14**	**15**	**16**	**17**	**18**	**19**
Position	−221	−210	−198	−153	−136	−106	−84	−24	−21	−12	3	8	22	24	37	46	69	121	158
1MD-T	7.0	7.0	7.0	3.0	—	7.0	7.0	3.0	—	3.0	3.0	1.0	3.0	—	3.0	3.0	3.0	1.0	1.0
2MD-T	6.0	9.0	3.0	—	—	—	3.0	—	—	—	—	3.0	6.0	9.0	—	—	—	12.0	3.0
3MD-T	23.0	29.0	23.0	6.0	3.0	6.0	19.0	1.0	6.0	13.0	—	6.0	3.0	26.0	3.0	6.0	6.0	26.0	19.0
4MD-T	7.0	1.0	7.0	—	—	3.0	14.0	14.0	1.0	21.0	3.0	17.0	3.0	14.0	1.0	14.0	7.0	28.0	31.0
5MD-T	1.0	1.0	17.0	7.0	7.0	3.0	13.0	3.0	—	7.0	3.0	17.0	3.0	23.0	3.0	3.0	3.0	2.0	17.0
1MD-C	7.0	7.0	3.0	—	—	—	7.0	7.0	3.0	7.0	3.0	3.0	—	7.0	7.0	3.0	7.0	17.0	17.0
2MD-C	14.0	21.0	21.0	11.0	—	—	25.0	7.0	7.0	21.0	14.0	25.0	18.0	18.0	7.0	14.0	7.0	32.0	29.0
3MD-C	13.0	19.0	6.0	3.0	—	6.0	16.0	6.0	6.0	1.0	6.0	6.0	6.0	13.0	6.0	6.0	3.0	29.0	1.0
4MD-C	2.0	2.0	2.0	—	3.0	7.0	3.0	1.0	7.0	27.0	7.0	2.0	3.0	17.0	3.0	1.0	13.0	47.0	33.0
5MD-C	12.0	18.0	9.0	3.0	6.0	6.0	21.0	12.0	9.0	18.0	6.0	18.0	3.0	18.0	3.0	3.0	6.0	3.0	21.0
1NC58	7.0	7.0	1.0	—	3.0	3.0	1.0	—	1.0	7.0	7.0	13.0	—	17.0	—	3.0	3.0	13.0	7.0
2NC58	7.0	7.0	3.0	—	7.0	3.0	17.0	—	1.0	3.0	3.0	1.0	—	13.0	7.0	—	—	13.0	13.0
3NC58	19.0	31.0	16.0	—	6.0	3.0	22.0	3.0	3.0	13.0	6.0	16.0	—	13.0	3.0	—	3.0	22.0	9.0
4NC58	9.0	13.0	9.0	3.0	—	3.0	16.0	13.0	9.0	13.0	3.0	16.0	—	19.0	3.0	9.0	9.0	34.0	25.0
5NC58	9.0	16.0	13.0	6.0	3.0	6.0	25.0	22.0	6.0	16.0	9.0	31.0	9.0	19.0	6.0	9.0	19.0	41.0	25.0

Form-deprived, contralateral control eyes after 1 week of recovery following 4 weeks of MD, and normal control eyes for 58 days. “—” means none detected.

**Figure 5:**
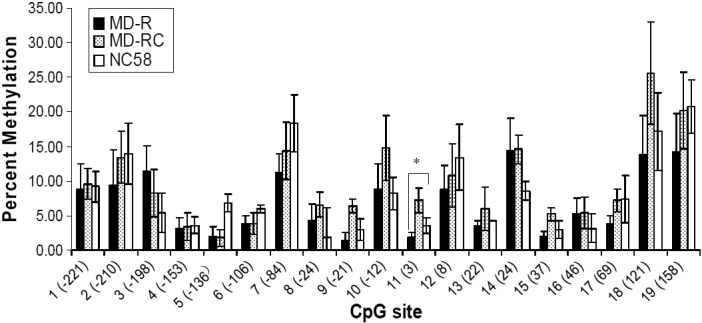
Methylation percentages of cytosine-phosphate-guanine (CpG) sites in the collagen type Iα1 promoter region in scleras of monocular deprivation and control eyes after four weeks of monocular deprivation and one week of recovery. Numbers in parentheses on the x-axis are the locations of the cytosine-phosphate-guanine (CpG) sites. Methylation at site 11 was significantly less in the monocular deprivation–recovery (MD-R) eyes than in the NC_58_ eyes. MD-R: after 7 days of recovery following 4 weeks of monocular deprivation, MD-RC: contralateral control eyes, NC_58_: age-matched normal control eyes for MD recovery, *, p<0.05.

In the MD-R eyes, the methylation percentages of the six CpG sites that were previously elevated (1, 3, 9, 14, 18, and 19) were similar to those of the MD-RC and NC_58_ eyes (Figure 5). Thus, the recovery from myopia was associated with a loss of DNA methylation at the CpG sites. The methylation percentage of CpG site 11 in the MD-R eyes was reduced significantly compared to the NC_58_ eyes (Figure 5).

### Downregulation of scleral *COL1A1* mRNA level during myopia

**Figure 6:**
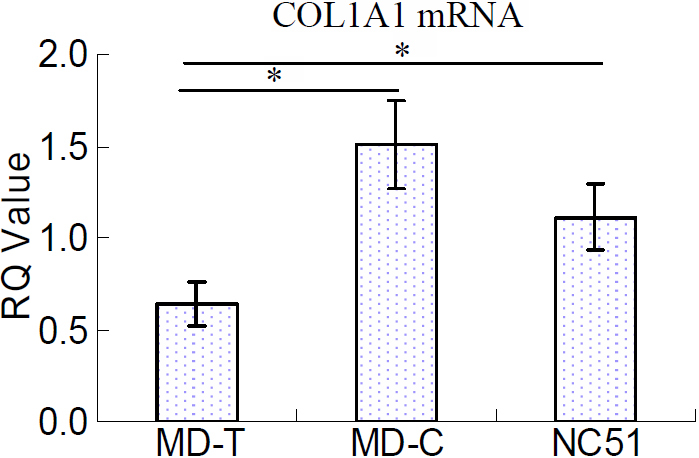
Scleral collagen type Iα1 mRNA levels in monocular deprivation and normal control eyes. There was significantly less collagen type Iα1 (*COL1A1*) mRNA in scleras from monocular deprivation–treated (MD-T) eyes compared to the MD-control (MD-C) eyes and the normal control (NC_51_) eyes. MD-C: contralateral control eyes, NC_51_: age-matched normal control eyes. *, p<0.05.

Scleral *COL1A1* mRNA levels were lower by 57% in the MD-T eyes than the MD-C eyes (p<0.05, Figure 6). Moreover, the *COL1A1* mRNA levels were 42% lower in the MD-T eyes compared to the normal control eyes (p<0.05, Figure 6).

## Discussion

Because of the large number of gene knockout and transgenic mouse models and the molecular tools available for studying them, murine models of induced myopia have advantages over other traditional species in some respects. Thus, mouse models have been increasingly used to study the molecular basis of myopia [21-23,28,29]. In our study, the MD-T eyes were significantly more myopic compared to the MD-C eyes and the normal control eyes (NC_51_). Similarly, the vitreous chamber depth was significantly increased at the MD-T eyes compared to the MD-C and NC_51_ eyes, results which were not different from other studies [29-32]. The axial length in MD-T eyes was significantly greater than in MD-C eyes, but not significantly greater than in NC_51_ eyes. There is a possible explanation for this apparent difference between the MD-C and NC_51_ eyes. There were great individual differences in axial length among the mice in each of the groups. This resulted in the detection of axial length differences between only the MD-T eyes and MD-C eyes of the same animals.

DNA methylation is known to inhibit gene expression in human cancer [16,17], murine cultured 3T3 cells, and F9 embryonal carcinoma cells [19]. The hypomethylation of CpG sites is also associated with overexpression of certain genes in cancer cells [18]. It is now known that the expression of *COL1A1* is controlled by many factors, including a change of DNA methylation status [33,34]. For instance, transformation of normal human lung fibroblasts by SV40, which is associated with increased DNA methylation, suppresses *COL1A1* gene expression [20].

Compared to the normal control eyes (NC_51_), the total methylation level in the CpG promoter sites for *COL1A1* increased significantly after four weeks of monocular form deprivation. Seven days after returning to normal vision, this level of methylation returned to the same levels as in the control eyes (NC_58_). The total methylation level in the MD-T eyes was significantly greater than in the NC_51_ eyes, but not the MD-C eyes, because the methylation level of some CpG sites of *COL1A1* in MD-C eyes also changed during myopia induction. Indeed, the methylation of sites 3, 8, 14, and 18 of *COL1A1* in the MD-C eyes tended to increase compared to the normal control eyes (NC_51_). This shows that form deprivation myopia in mice may also affect methylation of *COL1A1* in MD-C eyes, resulting in the absence of significant differences in total methylation between the MD-T and MD-C eyes.

Notably, methylation changes among MD-T, MD-C, and NC_51_ eyes were consistent with refraction changes of myopic eyes. During the period of form deprivation, the MD-C eyes also showed a myopic shift compared to the normal control eyes (NC_51_), albeit not significantly less than in the MD-T eyes. Barathi et al. [29] also found this phenomenon in form deprivation myopia in mice. The standard error of *COL1A1* gene expression in MD-C eyes was clearly larger than in the normal control eyes (NC_51_), indicating that some changes in gene expression may have occurred. These results suggest that in mice, unilateral form deprivation induces yoking effects in contralateral MD-C eyes. This phenomenon has also been observed in other animal models of myopia, such as the guinea pig [35], tree shrew [15,36], and rhesus monkey [37].

**Figure 7:**
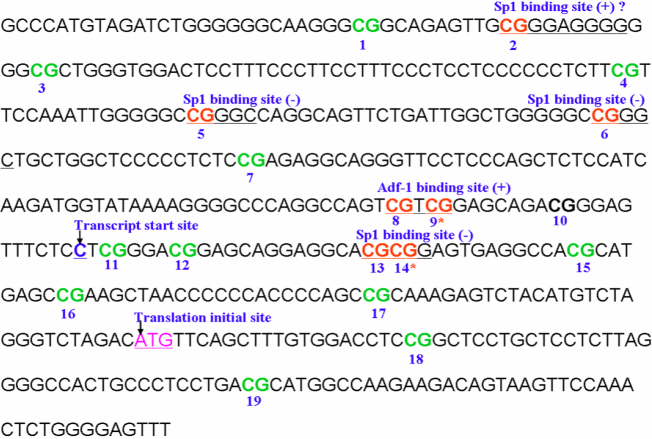
Amplification fragment of mouse collagen type Iα1 promoter region containing 19 cytosine-phosphate-guanine (CpG) sites. The online software P-Match 1.0 was used to predict transcription factor blinding sites. Site 9 is within the transcription factor Adf-1 binding site, and site 14 is within the transcription factor Sp1 binding site. Bold numbered cytosine-guanine (CGs) are cytosine-phosphate-guanine (CpG) sites. The notation “(+)” represents transcription factor binding to the positive strand of DNA, while “(-)” represents transcription factor binding to the negative strand of DNA. Moreover, “”? represents a partial match. Asterisks indicate significant differences between monocular deprivation–treated (MD-T) eyes and either the control or MD-recovery (MD-R) eyes.

CpG methylation site number 9 is within the binding site of transcription factor Adf-1, and CpG methylation site number 14 is within the binding site for transcription factor Sp1 (Figure 7). In *Drosophila*, Adf-1 activates the transcription of many genes [38-40]. In normal human dermal fibroblasts, Sp1 can activate the transcription of *COL1A1* [41] During MD, methylation of the 9th and 14th CpG sites may suppress *COL1A1* gene expression by altering Adf-1 and Sp1 binding. After MD recovery, those locations are demethylated, and allow the binding of Adf-1 and Sp1. The other four CpG sites, 1, 3, 18, and 19, which also became methylated during MD and were demethylated during recovery, are not located in transcription factor binding sites. The functions of these CpG sites are not currently known. The methylation of the CpG sites may have affected the structure of chromatin [42-44] or the binding of methyl-C-binding proteins [19] in the treated eyes of the MD group. Interestingly, the 11th CpG site, which underwent significant methylation and demethylation during treatment and recovery, is located near the transcription start site of *COL1A1*. The loss of CpG methylation at this site in the MD recovery eyes may promote the transcription of *COL1A1*, which suggests renewed transcription of *COL1A1* under these conditions.

Because of the small amount of DNA and mRNA present in the sclera, we used a pooling strategy for biologic analysis. For normal control animals, both eyes from each animal were pooled. For the MD-T group, the eyes from two animals were pooled. Thus, the normal control tissue samples were more homogenous than were the MD-T samples. This sample pooling and preparation method may have exaggerated the apparent statistical differences between these two groups. However, we also included the pooled MD-C eyes, which were the untreated contralateral controls to the MD-T eyes. Because these two groups were from the same animals, this comparison (MD-T eye versus MD-C eye) would fully address any possible exaggerated statistical differences between the MD-T and nontreated control eyes.

The *COL1A1* gene is speculated to be a susceptibility gene for high myopia, as it is located in MYP5 (17q21–22) of high myopia candidate locus and is downregulated during myopia in animal models [15-17]. However, until now, there has been no consensus with regard to its role in the development of myopia. One report links *COL1A1* polymorphisms with high myopia in Japanese subjects [11], but others do not confirm this [12,45-47]. Therefore, the association between *COL1A1* and human high myopia may not be completely attributed to the DNA sequences. Rather, epigenetic factors such as DNA methylation should also be considered. It is widely considered that the interplay of heredity and environmental factors is important in low and moderate myopia. Thus, epigenetic changes such as CpG methylation of *COL1A1* may play a more meaningful role in low and moderate myopia.

In summary, the frequency of methylation in CpG islands of the *COL1A1* promoter increased in the scleras of mouse MD eyes compared to control eyes. Associated with this DNA methylation, transcription of scleral *COL1A1* was suppressed. In eyes allowed to recover from MD, CpG methylation decreased and returned to a normal level, while the transcription of *COL1A1* increased. This finding suggests that DNA methylation of the *COL1A1* promoter/exon 1 may be linked with the inhibition of scleral collagen synthesis, which contributes to the development of myopia.
